# TSPO Deficiency Exacerbates GSDMD-Mediated Macrophage Pyroptosis in Inflammatory Bowel Disease

**DOI:** 10.3390/cells11050856

**Published:** 2022-03-02

**Authors:** Xue Zhang, Jingyi Han, Yi Xu, Menghua Cai, Fei Gao, Jiajia Han, Dongdong Wang, Yi Fu, Hui Chen, Wei He, Jianmin Zhang

**Affiliations:** 1 Department of Immunology, Research Center on Pediatric Development and Diseases, Chinese Academy of Medical Sciences, Key Laboratory of T Cell and Cancer Immunotherapy, Institute of Basic Medical Sciences, Chinese Academy of Medical Sciences and School of Basic Medicine, Peking Union Medical College, State Key Laboratory of Medical Molecular Biology, Beijing 100005, China; zhangxue921125@163.com (X.Z.); 13173056037@163.com (J.H.); xuyi2207@163.com (Y.X.); menghuacai@ibms.pumc.edu.cn (M.C.); gaofeipumc2015@163.com (F.G.); hanjiajia_1@163.com (J.H.); wddo1991@163.com (D.W.); fuyiabc@126.com (Y.F.); chenhui_1980@126.com (H.C.); 2 Department of Thoracic Surgery, Qilu Hospital, Cheeloo College of Medicine, Shandong University, Jinan 250012, China; 3 Changzhou Xitaihu Institute for Frontier Technology of Cell Therapy, Changzhou 213000, China

**Keywords:** TSPO, inflammatory bowel disease, GSDMD, pyroptosis

## Abstract

Background: the 18-kDa translocator protein (TSPO) is a mitochondrial outer membrane protein, and its expression tends to increase in response to inflammatory stimulation, rapidly. However, the role of TSPO in inflammation and pyroptosis is not yet clear. Here, we identified TSPO as a novel key regulator of pyroptosis. (2) Methods: TSPO knockout and DSS induced mouse inflammatory bowel disease (IBD) models were employed to assess the roles of TSPO in the pathogenesis of IBD. Primary peritoneal macrophages from TSPO knockout mice were applied to evaluate the mechanism of TSPO in cell pyroptosis. Conclusions: in response to inflammatory injury, TSPO expression is rapidly upregulated and provides a protective function against GSDMD-mediated pyroptosis, which helps us better understand the biological role of TSPO and a novel regulatory mechanism of the pyroptosis process.

## 1. Introduction

Inflammatory bowel disease (IBD) is an idiopathic inflammatory disease of the intestine that is characterized by continuous inflammation of the mucosal and submucosal layers of the colon and includes Crohn’s disease (CD) and ulcerative colitis (UC). Chronic intestinal inflammation usually involves the rectum first and gradually spreads to the entire colon, while it is accompanied by the activation of inflammasomes [1,2,3,4] and the release of cytokines, such as IL-1β, due to pyroptosis [5,6]. Pyroptosis is a form of programmed cell death associated with various inflammatory diseases through an excessive inflammatory response [7]. When cells are stimulated by inflammatory signals, such as LPS, the downstream inflammasome complex NLRP3 is activated through the classic or nonclassic inflammatory activation pathway and started caspase-1 shears GSDMD to form the N-terminal GSDMD fragment (p30). GSDMD (p30) has pore-forming toxicity and is the final effector protein of GSDMD-mediated pyroptosis [8,9]. Free GSDMD (p30) targets and inserts into the plasma membrane, forming pore-like structures with inner diameters of 10–20 nm [10,11]. Cellular inflammatory factors can leak through these pores to the extracellular space. If the pore-like structures continue to increase, there can be an imbalance in intracellular osmotic pressure, resulting in dramatic swelling of the cell, the eventual loss of membrane integrity, and even cell death [12,13].

In recent years, several studies have shown that pyroptosis effector proteins target not only the plasma membrane, but also the outer mitochondrial membrane [14]. Alterations in outer mitochondrial membrane permeability are usually essential initiating steps in cells in response to cell stress. Still, recent studies have shown that mitochondrial outer membrane permeabilization (MOMP) also promotes the development of pyroptosis [15,16]. The changes in MOMP cause substantial spillover of the reactive oxygen species (ROS) from the mitochondria into the cytoplasm, and intracellular NLRP3 and caspase-1 are activated. In this context, activated caspase-1 will further shear gasdermins, creating the activated pore-forming form, thus intensifying the formation of pores in the cell membrane and further expanding pyroptosis development [17]. From this perspective, mitochondrial stress plays an important role in developing pyroptosis. However, the exact position of mitochondria in pyroptosis needs to be further investigated.

The mitochondrion is the energy center of the cell. The 18-kDa translocator protein (TSPO) is one of the outer mitochondrial membrane proteins [18]. Recent studies revealed that TSPO could activate endoplasmic reticulum-associated protein degradation, inhibit autophagy, and increase the production of pro-inflammatory cytokines [19,20]. TSPO is closely associated with mitochondrial function, and our previous study showed that TSPO deletion resulted in more fragmented mitochondria, reduced mitochondrial membrane potential, and increased ROS levels in GL261 cells [21]. TSPO expression has also been reported to be highly upregulated in many inflammatory diseases and multiple tumor types, such as IBD and colon cancer. There is no abnormalities in the TSPO KO mice colon, and the lifespan of these TSPO KO mice was not affected [18,22,23]. However, the molecular mechanisms underlying TSPO-mediated inflammation and pyroptosis remain unknown.

In this study, we applied a DSS-induced IBD mouse model in TSPO knockout (KO) mice to investigate the role of TSPO in the pathogenesis of IBD. Our results revealed that TSPO played a crucial protective function in the development of IBD. TSPO deficiency causes more severe inflammatory damage and GSDMD-mediated macrophage pyrolysis.

## 2. Materials and Methods

### 2.1. DSS Stimulation Assay

The wild type (WT) and TSPO knockout (KO) mice were obtained from same littermates of het/het (TSPO^+/−^) breeding and co-housed throughout the whole experiment [18]. TSPO-KO and WT mice aged 6–8 weeks were selected as experimental mice. A murine model of DSS-induced acute colitis was performed as described previously [24]. In brief, the daily drinking water was replaced with 3% DSS solution every 4 days. The weight and fecal characteristics of mice were recorded every day, and the disease activity index (DAI) score was calculated. After 10 days of induction, the mice were euthanized for subsequent experiments.

### 2.2. Isolation of Murine LP Cells

Colonic LP cells were isolated from TSPO-KO and WT mice as described previously [25]. Briefly, colon tissues were isolated from the mice, opened longitudinally, and washed in cold PBS. The colon tissues were cut into 1 cm pieces and incubated in pre-digestive solution (HBSS without Ca^2+^/Mg^2+^, 5% FBS, 2 mM EDTA, 1000 × 2-ME) for 15 min at 37 °C, 250 rpm. Then, the tissues were cut into 5 mm pieces and was incubated in 2 mL digestive solution (HBSS, 5% FBS, 1 mg/mL collagenase IV, 0.1 mg/mL DNase I) for 20 min at 37 °C, 250 rpm. The supernatant was collected by a centrifugation of 600× *g* and the remaining tissues were added to 5 mL of digestive solution for 20 min at 37 °C, 250 rpm. The supernatant was collected and combined with the previous one before centrifugation at 300× *g* for 5 min [25]. After discarding the supernatant, the cell pellet was resuspended with PBS and filtered with 40 um cell sieves on ice.

### 2.3. Flow Cytometry

Isolated mouse colonic mononuclear cells or mesenteric lymph node cells were placed into a 1.5 mL EP tube before washing once with 1 × PBS solution. Then, 1 mL of 1% BSA blocking solution was added to each tube, and the cells were spun again at 300× *g* for 5 min to remove the supernatant before the addition of 2 μL of each antibody and incubated for 30 min at 4 °C as describe previously [21].

### 2.4. Isolation and Culture of Murine Macrophages

Both WT and TSPO-KO mice were intraperitoneally injected with a 3% Brewer thioglycollate medium and euthanized by rapid cervical dislocation after 3 days of inflammatory responses. Cold PBS was used to lavage the abdominal cavity, and the peritoneal irrigation fluid was collected and centrifuged at 300× *g* and 4 °C for 10 min.

### 2.5. Pyroptosis Stimulation Assay

The complete medium was replaced with serum-free medium 4 h after cell isolation from mice, and the cells were starved for 12 h. LPS at a final concentration of 1 μg/mL was added for pre-stimulation for 3 h before the boost stimulation with ATP at a final concentration of 5 mmol/L, as described previously [26]. The cells or culture supernatants were collected and analyzed at different time points.

### 2.6. LDH Assay

A CytoTox 96^®^ Non-Radioactive Cytotoxicity Assay kit (Madison, WI, USA) was applied to assess LDH release by mouse macrophages after pyroptosis as previously described [27]. In brief, mouse primary peritoneal macrophages were plated in 96-well plates and incubated at 37 °C and 5% CO_2_ for 12 h. Ten microliters of total lysis solution buffer were added to untreated cells 30 min before the end of pyroptosis stimulation as a control for maximum LDH release. After pyroptosis stimulation, the plate was centrifuged at 250× *g* for 4 min, and 50 μL of the supernatant was transferred into a new 96-well plate. After 50 μL of reaction substrate buffer was added to each well, the plate was incubated for another 30 min in the dark at room temperature (RT). Then, 50 μL of stop buffer was added. The optical density (OD) value was measured immediately.

### 2.7. Western Blotting

After pyroptosis stimulation, the supernatant was removed from the cells, and the cells were lysed in EDTA-free RIPA buffer containing a protease inhibitor (Thermo Scientific, Waltham, MA, USA) on ice for 30 min. Cells were seeded in a 24-well plate (5 × 10^5^ cells/well) with 300 μL culture medium. A total of 25 μL of supernatant per sample was used for Western blotting. Both the supernatant and cell lysate were centrifuged at 1000× *g* and 4 °C for 15 min. SDS-PAGE was used to resolve proteins by molecular weight. Proteins in both the supernatant and cell lysates were incubated with primary antibodies for 10 h at 4 °C followed by incubation with anti-mouse or anti-rabbit secondary antibodies (Invitrogen, Waltham, MA, USA) for 2 h at room temperature. The signal was developed with SuperSignal West Pico Chemiluminescent Substrate (Thermo Scientific, Waltham, MA, USA) and a CliNX ChemiScope 3400 (CliNX Science Instruments Co., Ltd., Shanghai, China).

### 2.8. Immunofluorescence Assay

Cells were seeded on microscope cover slides (Fisherbrand, Waltham, MA, USA) and incubated overnight. After the treatments, the cells were washed three times with PBS and fixed with 4% formaldehyde for 15 min. Then, the cells were permeabilized with 0.25% Triton X-100 in PBS for 10 min and blocked with goat serum for blocking (Boster, Pleasanton, CA, USA) for 1 h at room temperature. Cells were stained with primary antibodies for 10 h at 4 °C and then incubated with Alexa Fluor Plus 555 goat anti-rabbit IgG secondary antibodies (Invitrogen) at a dilution of 1:1000 for 2 h at RT. Images were taken on a Zeiss LSM 710 confocal microscope.

### 2.9. Multiplex Immunohistochemistry Assay

Murine colon sections were prepared for multiplex immunohistochemistry staining as described previously [28]. Briefly, the sections were deparaffinized through xylenes and rehydrated through decreasing graded alcohol. Endogenous peroxidase was removed by incubation in 3% H_2_O_2_ for 15 min. and 1X Tris-EDTA pH 8.0 was used for antigen retrieval in a microwave oven. A hydrophobic pen was used to circle tissue sections before blocking with goat serum for 10 min at RT. Then, the sections were incubated with primary antibodies (anti-PBR or anti-GSDMD) at 37 °C for 2 h. Sections were then incubated with Rb HRP-Polymer (ZSGB-BIO) for 10 min at RT. Incubation with Opal 7 color manual kit (Akoya) for 2 min was followed by washing in 1X TBST. Images were taken on AKOYA Vectra Polaris microscope.

### 2.10. Co-Immunoprecipitation (Co-IP) Assay

Mouse macrophages were isolated and seeded at 1 × 10^6^ cells/mL in 10 cm dishes and incubated at 37 °C and 5% CO_2_ overnight. After pyroptosis induction, the cells were washed with PBS three times and lysed in 500 μL of NP-40 IP lysis buffer containing 1% protein phosphatase inhibitor (Abcam) on ice. Cells were collected and centrifuged at 12,000× *g* rpm and 4 °C for 15 min. The supernatant was transferred into new tubes and incubated with primary antibodies for 10 h at 4 °C. Dynabeads^™^ Protein G for immunoprecipitation (Invitrogen) was added to each tube and incubated at RT for 15 min. After incubation, the magnetic bead-Ab-Ag complex was gently washed 5 times, the supernatant was removed, and the bead-Ab-Ag complex was lysed in SDS sample buffer, boiled for 5 min, and analyzed by SDS-PAGE.

### 2.11. Antibodies

Rabbit monoclonal Anti-ASC (AG-25B-006-C100, AdipoGen, San Diego, CA, USA), mouse monoclonal anti-NLRP3 (AG-20B-0014-C100), rabbit monoclonal anti-caspase-1 (p20) (AG-20B-0042-C100), Rabbit monoclonal anti-GSDMD (ab209845, ABCAM, Cambridge, UK), rabbit monoclonal anti-PBR (ab109497, ABCAM), mouse Antiβ-actin (SJ190a9b68548, Sigma-Aldrich, St. Louis, MO, USA), rabbit monoclonal anti-IL-1β (12242, CST, Danvers, MA, USA), rabbit monoclonal anti-Iba1 (PA5-21274, Invitrogen), mouse monoclonal anti-Iba1 (019-19741, WAKO, Osaka Japan), anti-cytochrome C antibody (ab90529, Abcam) FITC anti-mouse CD45 antibody (103108, BioLegend, San Diego, CA, USA), APC anti-mouse F4/80 antibody (123116, BioLegend), PE/Cyanine7 anti-mouse CD86 antibody (105014, BioLegend), PE anti-mouse/human CD11b antibody (101208, BioLegend).

### 2.12. Statistical Analysis

Results are expressed as mean ± S.E.M. Statistical significance was determined by Student’s *t*-test, two-sided. Differences of statistical analysis at * *p* < 0.05, ** *p* < 0.01, *** *p* < 0.001.

## 3. Results

### 3.1. TSPO-KO Mice Developed More Severe Colitis in DSS-Induced IBD Mice

**Figure 1:**
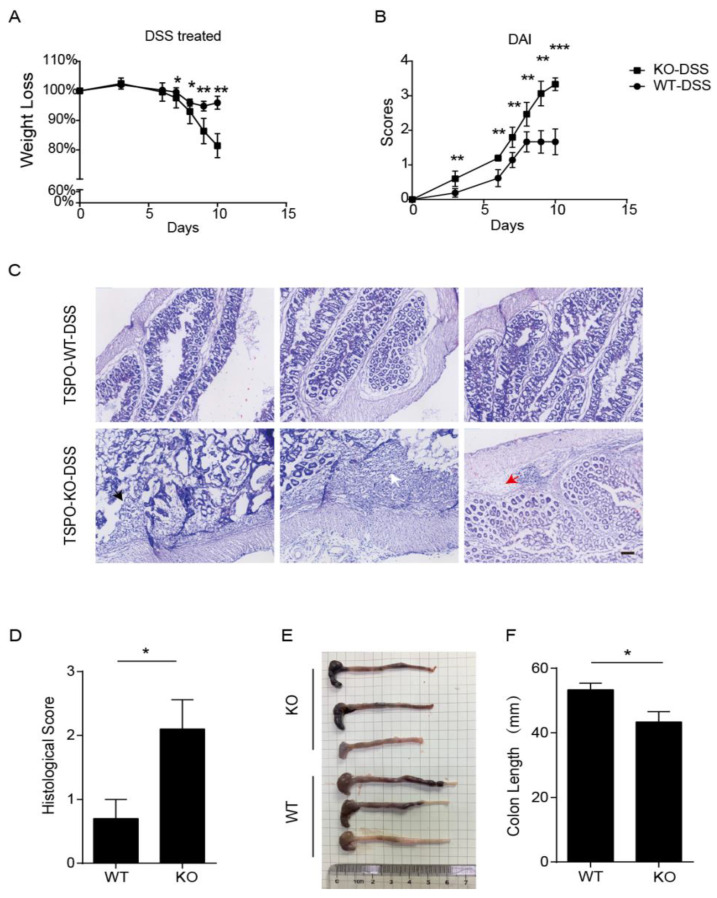
TSPO deletion results in more severe colitis in DSS-induced IBD mice. (**A**) Weight loss of TSPO KO and WT mice after DSS treatment (WT, n = 7; KO, n = 5). (**B**) Disease activity index (DAI) scores of TSPO and WT mice after DSS treatment (WT, n = 7; KO, n = 5). (**C**) HE staining of TSPO KO and WT mouse colons showed ulceration (black arrow), areas of strong transmural inflammation (white arrow), edematous submucosa (red arrow), and other inflammatory phenotypes in the colons of TSPO KO mice; the scale bar represents 100 μm. (**D**) Inflammatory histological scores of TSPO KO and WT mice (WT, n = 5; KO, n = 5). (**E**) Colon length of TSPO KO and WT mice. (**F**) Colon length statistics of TSPO KO and WT mice (WT, n = 5; KO, n = 5). Data expressed as mean ± s.e.m., Student’s *t*-test, two-sided. * *p* < 0.05, ** *p* < 0.01, *** *p* < 0.001.

To characterize the possible links between intestinal immunity and TSPO, we applied TSPO-KO and TSPO wild type (TSPO-WT) mice to create DSS-induced acute and chronic colitis murine models. TSPO-WT and TSPO-KO mice were euthanized after receiving 2.5% DSS in their drinking water for 10 days, and the disease activity index (DAI) scores of all mice were evaluated each day. Compared with WT mice, TSPO-KO mice had more significant body weight loss (Figure 1A). The increased DAI scores were more pronounced in TSPO-KO mice than in TSPO-WT mice (Figure 1B), indicating that weight loss, blood stool, and soft stools were more severe in KO mice than in WT mice. Both WT and KO mice were sacrificed on day 10, and we isolated the colons. The colon lengths of TSPO-KO mice were reduced compared with those of WT mice, and TSPO-KO mouse colons exhibited more severe mucosal congestion (Figure 1C,D), which indicated more severe colonic injury in TSPO-KO mice than in WT mice. In addition, we collected mouse colon tissue for pathological examinations. We found that the colons of TSPO-KO mice more frequently contained colonic mucosal erosions and ulcers, goblet cell loss, inflammatory hyperproliferative primary mucosal cell clusters, and even transmural infiltration (Figure 1E). Inflammation-associated histological scores were significantly higher in TSPO-KO mice than in WT mice (Figure 1F). Taken together, these results suggest that TSPO deficiency results in more severe colitis in a DSS-induced IBD mouse model.

### 3.2. TSPO Deficiency Results in More Pro-Inflammatory M1 Macrophages in the Colon

**Figure 2:**
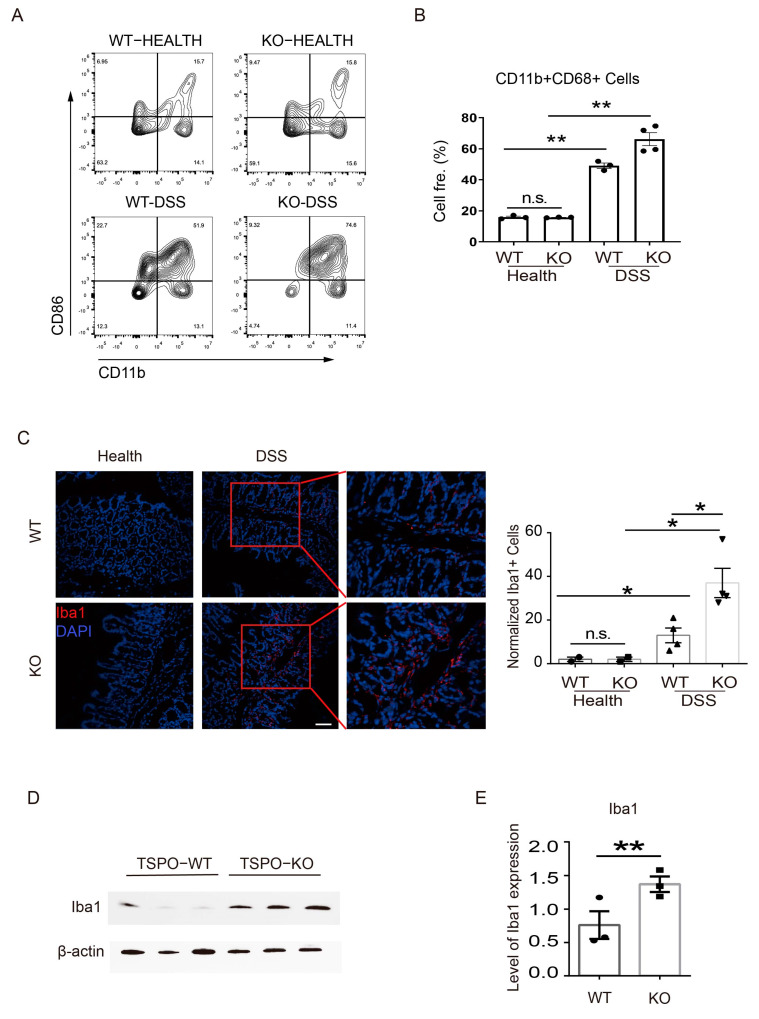
TSPO KO mice exhibited more pro-inflammatory M1 macrophages in the colon. (**A**) Flow cytometry analysis of the proportion of M1 type macrophages in mouse colon mononuclear cells from WT (healthy n = 3. DSS n = 3) and KO mice (healthy n = 3. DSS n = 4). (**B**) The statistics of the proportion of macrophages in mouse colon lymphocytes are shown in panel A. (**C**) Left, Iba1 immunofluorescence staining of frozen mouse colon sections from WT and KO mice was visualized by laser confocal microscopy. Red: Iba1. Blue: DAPI. The scale bar: 50 μm. Right, quantification of the expression levels of Iba1 as shown in left. (**D**) After DSS treatment, Iba1 expression levels in lymphocyte lysates from TSPO KO and WT mice were analyzed by Western blots. (**E**) Quantification of the expression levels of Iba1 as shown in Panel D. Data expressed as mean ± s.e.m., Student’s *t*-test, two-tailed. * *p* < 0.05, ** *p* < 0.01. n.s. means not significant.

To decipher the mechanisms leading to severe DSS-induced colitis in TSPO-KO mice, we analyzed local lymphocyte compositions in the colons of TSPO-KO and WT mice under physiological and pathological conditions. Flow cytometry analysis showed no difference between the proportion of CD11b^+^/CD86^+^ M1 macrophages among lamina propria (LP) cells in either healthy TSPO-KO or WT mice (Figure 2A,B). However, after 10 days of DSS administration, the proportion of CD11b^+^/CD86^+^ M1 macrophages in both KO and WT mice was obviously increased, and the proportion of M1 type macrophage in KO mice was slightly higher than that in WT mice (*p* = 0.057) (Figure 2A,B). These findings suggest that TSPO deficiency causes more pro-inflammatory M1 macrophages in the colon, contributing to severe DSS-induced colitis in TSPO-KO mice.

To further verify the flow cytometry results, immunofluorescence analyses were performed to examine macrophage infiltration in the colonic intestinal villous tissues of TSPO-KO and WT mice. The results showed that there were very few Iba1 + macrophages in the colonic tissues under physiological conditions from either WT mice or TSPO-KO mice (Figure 2C). Still, in mice with DSS-induced acute colitis, the infiltration of Iba1 + macrophages was significantly increased in both TSPO-KO and WT mice. The number of Iba1 + macrophages infiltrated in the colonic intestinal villi in TSPO-KO mice was much higher than that in WT mice (Figure 2C). This result was also verified by Western blot analysis, which showed a significant upregulation of Iba1 expression in colonic LP cells from TSPO-KO mice compared to WT mice (Figure 2D,E). Taken together, these results revealed that in DSS-induced mice, macrophages extensively infiltrated the colonic villi and exerted their pro-inflammatory effects. In contrast, macrophage infiltration in TSPO-KO mice was much higher than in WT mice. These excessively infiltrating macrophages in the intestinal villi were probably the effector cells of the worsened symptoms of acute colitis in TSPO-KO mice.

### 3.3. TSPO Deficiency Promotes Pyroptosis in Peritoneal Macrophages

**Figure 3:**
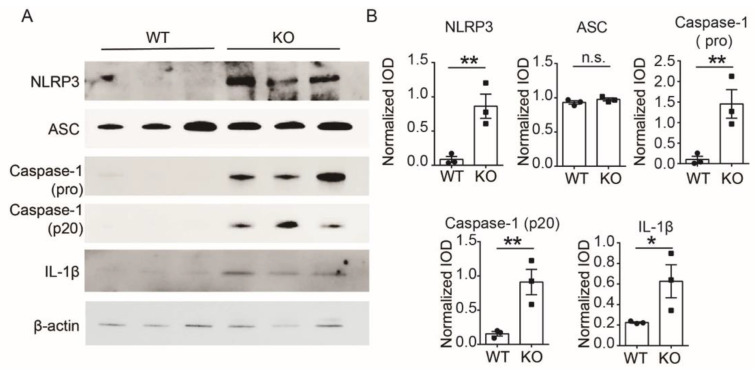
TSPO deficiency promotes pyroptosis in macrophages. (**A**) The expression of NLRP3, caspase-1, caspase-1 (P20), and the pro-inflammatory cytokine IL-1β in TSPO KO and WT mouse colon mononuclear cells was increased after DSS treatment; however, there was no significant difference between the expression of ASC in TSPO KO and WT colon mononuclear cells. (**B**) Statistical analysis of the expression of NLRP3, caspase-1, caspase-1 (P20), and the pro-inflammatory cytokine IL-1β in TSPO KO and WT mouse colon mononuclear cells after DSS treatment. Three completely independent experiments were conducted. Data are shown as mean ± s.e.m., Student’s *t*-test, two-tailed. * *p* < 0.05, ** *p* < 0.01. n.s. means not significant.

The finding that macrophage infiltration was significantly increased in DSS-induced TSPO-KO mice prompted us to determine whether TSPO deficiency affected the macrophage response to inflammation. We then sorted colonic LP cells from DSS-induced WT and TSPO-KO mice. We observed that the levels of inflammasome-related molecules such as NLRP3, caspase-1, and cleaved caspase-1 were significantly higher in TSPO-KO mice than in WT mice. In contrast, there was no significant difference in the expression of ASCs (Figure 3A,B) and GSDMD (Supplementary Figure S1A) between WT and KO cells. These data indicate that the absence of TSPO caused a more severe inflammasome-related inflammatory response. Notably, the expression of the inflammasome- and pyroptosis-associated pro-inflammatory factor IL-1β was significantly enhanced in TSPO-KO mice (Figure 3A,B). Overall, the upregulation of inflammasome-related proteins in KO cells suggests that TSPO is involved in regulating inflammasome pathways and that TSPO deficiency leads to excessive NLRP3 activation, which could be a key factor in inducing downstream pyroptosis.

### 3.4. TSPO Deficiency Promotes GSDMD-Induced Pyroptosis in Mouse Peritoneal Macrophages

**Figure 4:**
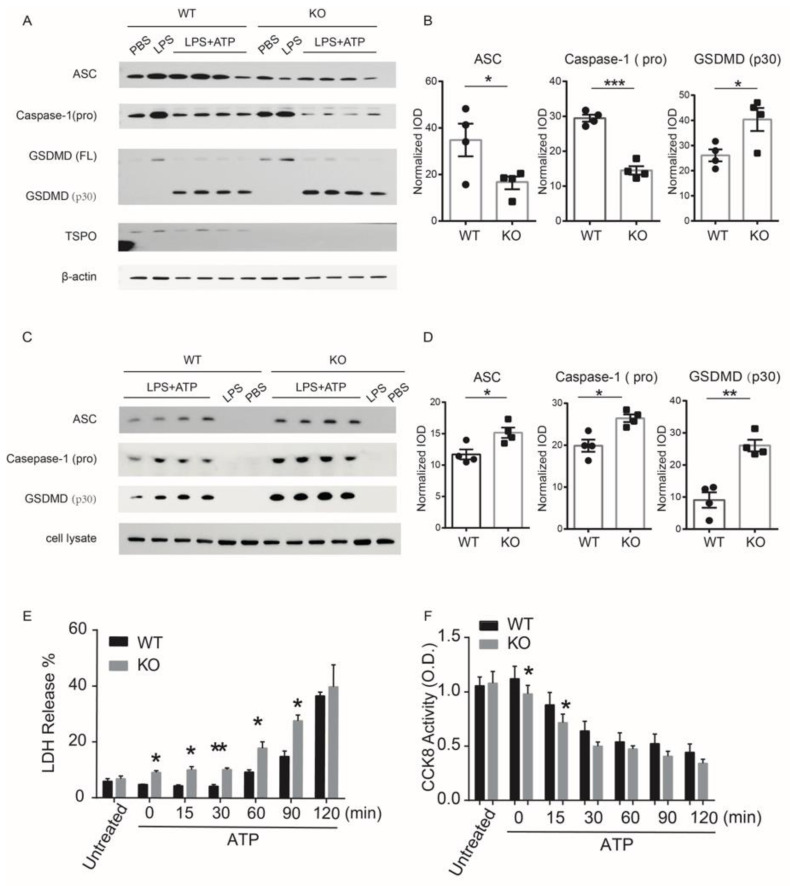
TSPO deficiency causes enhanced GSDMD-induced pyroptosis in macrophages. (**A**) The expression levels of ASC, caspase-1, and GSDMD (P30) in mouse peritoneal macrophages after inflammatory stimulation. (**B**) Statistical analysis of the expression levels of ASC, caspase-1, and GSDMD (P30) in mouse peritoneal macrophages after inflammatory stimulation. (**C**) The expression levels of ASC, caspase-1, and GSDMD (P30) in mouse peritoneal macrophage culture supernatant after inflammatory stimulation. (**D**) Statistical analysis of the expression levels of ASC, caspase-1, and GSDMD (P30) in mouse peritoneal macrophage culture supernatant after inflammatory stimulation. Three completely independent experiments were conducted. (**E**) The level of LDH release were measured to detect the proportion of macrophage injury and death after inflammatory stimulation with LPS and ATP at different time points as indicated. Untreated, untreated control group. (**F**) CCK-8 toxicity test was conducted to detect the activity of peritoneal macrophages after inflammatory stimulation. Untreated: untreated control group. Data expressed as mean ± s.e.m., Student’s *t*-test, two-tailed. * *p* < 0.05, ** *p* < 0.01, *** *p* < 0.001.

Next, we sought to determine whether TSPO deficiency affected the levels of pyroptosis-related proteins during the process of pyroptosis. We performed Western blotting to measure inflammasome- and pyroptosis-related proteins in these cells and found that the levels of the pyroptosis-related proteins caspase-1 and ASC in the lysates of these cells were significantly reduced in TSPO KO macrophages compared to WT macrophages (Figure 4A,B). The level of caspase-1 (p20) was also increased in KO cells (Supplementary Figure S1B). The massive reduction in caspase-1 and ASC indicates an increase in inflammasome formation and a high level of GSDMD cleavage in KO cells. Correspondingly, the level of GSDMD (P30), an essential protein responsible for pyroptosis, was also significantly increased (Figure 4A,B). We also found that the levels of caspase-1 and GSDMD expression in unstimulated macrophages from WT and TSPO-KO mice were at the same level (Figure 4A and Supplementary Figure S1C).

We hypothesized that the reduction in inflammasome-related proteins might be due to the loss of cell membrane integrity caused by pyroptosis, which leads to the release of a large number of related proteins. We then measured protein levels in the cell supernatant. We found that the levels of inflammasome- and pyroptosis-related proteins caspase-1, ASC, and GSDMD in the TSPO KO cell supernatant were significantly higher than those in the WT cell supernatant (Figure 4C,D), indicating that the absence of TSPO causes rapid shearing and the release of inflammasome- and pyroptosis-related proteins.

To explore the effect of TSPO deletion on the inflammatory response of macrophages, peritoneal macrophages were isolated from TSPO-KO mice and WT mice. After stimulation with LPS and ATP to induce pyroptosis, the levels of lactate dehydrogenase (LDH) in the culture supernatant were significantly increased in KO cells compared with those in WT cells at different time points (Figure 4E). The numbers of viable KO cells were also decreased significantly at the early time points after stimulation compared with those of WT cells (Figure 4F). In the induction of pyroptosis with LPS and nigericin, we also observed enhanced pyroptosis of KO cells (Supplementary Figure S1D). These results taken together indicate that TSPO deficiency promotes peritoneal macrophages to undergo pyroptosis in the early stage of inflammatory response.

### 3.5. TSPO Inhibits Pyroptosis by Interacting with Mitochondria-Targeted GSDMD

**Figure 5:**
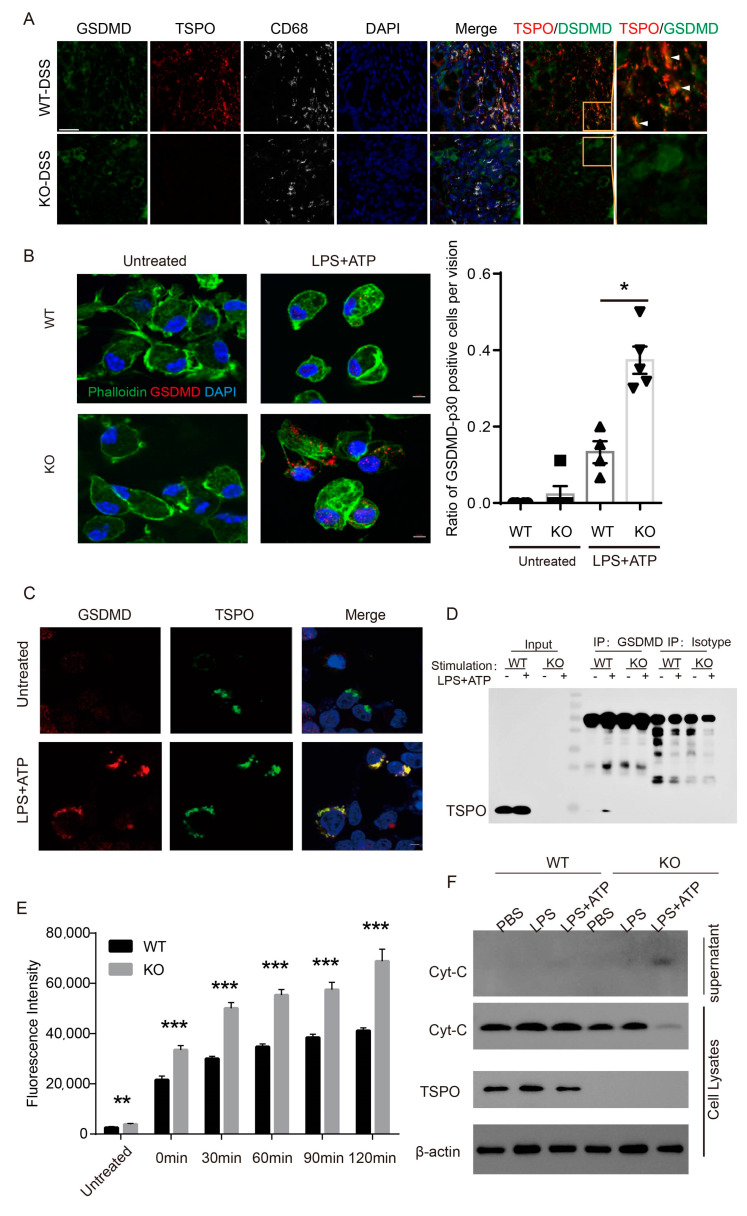
TSPO interacts with GSDMD during pyroptosis. (**A**) Multiplex immunohistochemistry assay of TSPO, GSDMD, and CD68 expression in colon tissue from WT and KO mice. Red: TSPO, white: CD68, green: GSDMD; blue, DAPI. White arrows represent for colocalization of TSPO and GSDMD. Scale bars, 20 μm. (**B**) Left, the subcellular localization of GSDMD in TSPO KO and WT mouse peritoneal macrophages was observed by laser confocal microscopy under an oil microscope. GSDMD was labeled with an anti-GSDMD antibody (red), the cytoskeleton was labeled with phalloidin (green), and the nucleus was labeled with DAPI (blue). Scale bar, 5 μm; right, quantification of GSDMD-p30 positive cells per vision of LPS + ATP activated macrophages. n = 5. (**C**) The subcellular localization of GSDMD and TSPO in HEK-293T cells was observed by laser confocal microscopy under an oil microscope. GSDMD was labeled with anti-GSDMD antibody (red), TSPO was labeled with GFP (green), and the nucleus was labeled by DAPI (blue). (**D**) IP verified the interaction between TSPO and GSDMD in pyroptotic macrophages. (**E**) Microplate assay for detection of H2DCFDA to quantitatively assess reactive oxygen species (ROS) levels in TSPO KO mouse peritoneal macrophages with Ex = 485 nm and Em = 535 nm. (**F**) Western blot assay to examine the cytochrome C in both cell lysate and supernatant of WT and KO cells. Data expressed as mean ± s.e.m., Student’s *t*-test, two-sided. * *p* < 0.05, ** *p* < 0.01, *** *p* < 0.001. Three independent experiments were conducted.

Next, we sought to determine whether TSPO interacts with GSDMD during pyroptosis. We performed multiplex immunohistochemistry to examine the colocalization of TSPO with GSDMD in mouse colon tissues. The result showed an obvious co-localization of TSPO and GSDMD in colon tissue from DSS-treated mouse (Figure 5A).

To further explore the key role of TSPO in pyroptosis, we applied a fluorescence confocal microscope to examine the dynamic changes in TSPO-deficient cells during pyroptosis. Immunofluorescence staining of GSDMD showed that no visible GSDMD expression was observed in either WT macrophages or TSPO KO cells under physiological conditions. This may be in that in steady state the pore-forming GSDMD-p30 (GSDMD-N domain) is “embosked” by its GSDMD-C domain [29], resulting in no antibody staining signal in immunofluorescent assay. However, after LPS plus ATP treatment, there were more GSDMD puncta in TSPO KO macrophages than in WT macrophages (Figure 5B). To examine whether TSPO interacted with GSDMD, TSPO-GFP expression plasmids were transfected into 293T cells. The results showed that TSPO and GSDMD were specifically colocalized after LPS plus ATP treatment, indicating that TSPO may interact with GSDMD during pyroptosis (Figure 5C).

To verify this possibility, we performed a Co-IP experiment using peritoneal macrophages. The results showed that although no detectable interaction between TSPO and GSDMD was observed under steady state, after treatment with LPS plus ATP to induce pyroptosis, we identified a GSDMD signal interacting with TSPO (Figure 5D and Supplementary Figure S1E).

Previous studies have shown that the pore-forming fragment of GSDMD (p30) can target not only the outer cell membrane but also the outer mitochondrial membrane, thus changing the MOMP. TSPO serves as an outer mitochondrial membrane protein and maintains the structural integrity of mitochondria. Thus, we sought to determine whether the interaction between TSPO and GSDMD played a role in MOMP stability and ROS release in the context of pyroptosis. We used a DCFDA probe to measure intracellular ROS levels and found that in resting physiological cells, intracellular ROS levels in TSPO-KO peritoneal macrophages were slightly higher than those in WT cells, consistent with our previously published observations. After treatment with LPS plus ATP to induce pyroptosis in these cells, intracellular ROS levels in both TSPO-KO and WT cells increased significantly. However, compared to WT cells, a significant increase in ROS levels was observed in TSPO-KO cells, indicating a substantial impairment of mitochondrial MOMP in TSPO-KO cells (Figure 5E). To assess mitochondrial damage, we measured the release of cytochrome C from the mitochondria in both cell lysates and supernatant of WT and KO cells. In steady state, no cytochrome C was detected in the supernatant of WT and KO cells. However, after the induction of pyroptosis with LPS and ATP, the cytochrome C in TSPO KO cells significantly released to the supernatant (Figure 5F), indicating a severe mitochondrial damage in KO cells during pyroptosis.

## 4. Discussion

Pyroptosis represents a form of programmed cell death that is triggered by pro-inflammatory signals and associated with inflammation. Organisms fight bacterial infections, local inflammation, and cancer through pyroptosis. A small degree of pyroptosis helps the body to actively clear invading pathogens and maintain homeostasis, while excessive pyroptosis releases large amounts of pro-inflammatory cytokines. Therefore, maintaining the level of pyroptosis within a normal range and controlling the intensity of pyroptosis is of great importance for treating inflammatory diseases. In response to inflammatory signals, macrophages are activated through toll-like receptors (TLRs) on the cell surface. Subsequently, the downstream inflammatory protein NLRP3 is activated to recruit the inflammatory junction protein ASC and the activated effector enzyme caspase-1 to assemble into inflammasomes. Activated caspase-1 hydrolyzes intracellular GSDMD into two parts: the N-terminal effector fragment (p30) with membrane pore-forming activity and the C-terminal fragment (GSDMD CT) with inhibitory pore-forming activity. GSDMD (p30) targets phospholipid structures with negatively charged head groups on the plasma membrane and inserts into the plasma membrane to form a multimeric pore-like structure, leading to an imbalance in osmotic pressure inside and outside the cell, vacuolization of the plasma membrane, and eventually cell death [30,31]. Therefore, inhibiting GSDMD hydrolysis and its pore-forming activity in the context of pyroptosis has become a novel therapeutic strategy against inflammatory diseases, such as IBD.

Recent studies have shown that pyroptosis is often accompanied by impaired mitochondrial function. The pore-forming active fragment GSDMD (p30) targets not only the cell membrane but also the outer mitochondrial membrane, thereby disrupting the continuity of the outer mitochondrial membrane and causing a substantial increase in MOMP [32,33]. The mitochondrial damage that accompanies pyroptosis, if not rapidly controlled, will lead to the release of large amounts of ROS from the mitochondria into the cytosol, activating the inflammatory vesicle complex and further inducing a shift toward inflammation. Therefore, normal mitochondrial function plays a critical role in maintaining the stability of the intracellular environment, inhibiting inflammasome activation and resisting pyroptosis.

TSPO, an outer mitochondrial membrane protein, is involved in a variety of biological processes, such as endoplasmic reticulum-associated protein degradation, autophagy, pro-inflammatory cytokine production, angiogenesis, and tumors [34]. TSPO deficiency causes an imbalance in mitochondrial metabolic function, decreased oxidative phosphorylation and ATP production, increased intracellular ROS levels, and a shift in cellular metabolism from oxidative phosphorylation to the glycolytic pathway, resulting in increased angiogenesis, glioma growth, and increased malignancy [21]. However, some independent laboratories have constructed TSPO global and conditional knockout models and reported that there were no abnormalities in TSPO KO mice colon, and the lifespans of TSPO KO mice were not affected [18,22,23].

In this study, we investigated the roles of TSPO in pyroptosis and inflammatory disease by using a DSS-induced acute colitis mouse model. TSPO-KO mice exhibited more severe colonic inflammatory injury than WT mice. The number of macrophages, especially pro-inflammatory M1 macrophages, in the colons of TSPO-KO mice was increased. Previous studies have demonstrated that in healthy mice, intestinal macrophages are mostly M2 type macrophages. However, in IBD patients, intestinal macrophages were mainly derived from M1 type (CD86+) macrophages recruited from the abdominal cavity [35]. Thus, our findings suggest that TSPO deficiency results in a phenotypic change in macrophages from M2 to M1 and promotes the inflammatory response, which was also confirmed by the results showing the expression of NLRP3, caspase-1, and IL-1β was significantly increased in TSPO-KO colonic LP cells.

Next, we found that TSPO-KO mouse peritoneal macrophage death was significantly higher than that of WT cells after pyroptosis signal induction, indicating that TSPO plays a protective role against pyroptosis. Then, we conducted a series of in vitro experiments using mouse peritoneal macrophages to investigate the relationship between TSPO and pyroptosis. LDH release assay, CCK-8 cytotoxicity assay, and Western blotting verified that TSPO deficiency resulted in enhanced pyroptosis. Interestingly, the expression of GSDMD (p30), a key effector protein of pyroptosis, was significantly upregulated, and the levels of caspase-1 and ASC were decreased in TSPO KO macrophages after pyroptosis signal induction. In contrast, the expression levels of GSDMD (p30), caspase-1, and ASC were increased in the cell culture supernatants, suggesting that the decrease in caspase-1 and ASC was probably due to the leakage of these two proteins from the cell due to pyroptosis. Moreover, immunofluorescence assays verified that TSPO-KO cells exhibited more GSDMD puncta accumulation than WT cells. Thus, our findings demonstrate that TSPO protects against pyroptosis by reducing the expression of GSDMD and its cleavage.

TSPO expression is rapidly upregulated in response to inflammatory stimulation. We observed that there is a colocalization between TSPO and GSDMD in DSS treated mice colon. Here, we performed cellular immunofluorescence and endogenous Co-IP assays and observed that under resting conditions, TSPO hardly interacted with GSDMD in macrophages. Still, two proteins exhibited a significant interaction when stimulated by pyroptosis signals. This finding reveals that the rapid upregulation of TSPO in response to inflammatory stimulation provides a protective function and inhibits cell death by interacting with GSDMD. This conclusion was also confirmed by the findings that TSPO-KO cells displayed a significant increase in ROS levels and a significant impairment of mitochondrial MOMP in response to pyroptosis-inducing signals.

In summary, our major finding in this study is the identification of TSPO as a novel key regulator of pyroptosis. In response to inflammatory injury, TSPO expression is rapidly upregulated. It provides a protective function against GSDMD-mediated pyroptosis, which can be an interpretation of the mechanism underlying the quick upregulation of TSPO expression in response to the inflammatory response in the field.

## Data Availability

Not applicable.

## Supplementary Materials

The following supporting information can be downloaded at: https://www.mdpi.com/article/10.3390/cells11050856/s1, Figure S1: TSPO deficiency does not affect the expression of pyroptosis-related proteins in steady macrophages.
