# Supplementary material for: TSPO Deficiency Exacerbates GSDMD-Mediated Macrophage Pyroptosis in Inflammatory Bowel Disease
Source: Cells. 2022 Mar 2;11(5):856. doi: 10.3390/cells11050856 (PMC8909696; doi:10.3390/cells11050856)
Supplement: Supplementary file 1 [file cells-11-00856-s001.zip › cells-1486829-supplementary.pdf]

Supplementary Material

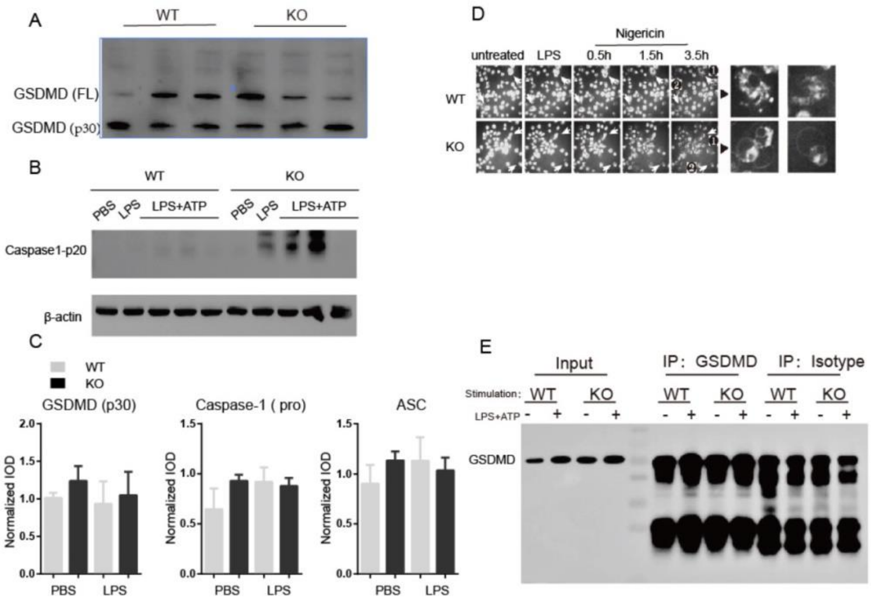

Figure S1. (**A**) The expression of GSDMD in TSPO KO and WT mouse colon mononuclear cells. (**B**) The expression of Caspase-1(p20) in TSPO KO and WT mouse peritoneal macrophages. (**C**) The quantification of GSDMD, Caspase-1 and ASC levels in unstimulated and only LPS treated macrophages. (**D**) Mouse peritoneal macrophages were stained with ER tracker, and immunofluorescence long-term imaging recorded inflammasome activation-induced pyroptosis. (**E**) Western blot to detect the amount of GSDMD proteins in the Co-IP assay as shown in Figure 5D.
